# Systematic review and network meta-analysis of performance of the Sapporo criteria, the Revised Sapporo criteria, and the 2023 ACR/EULAR APS classification criteria for patients with antiphospholipid syndrome: A protocol

**DOI:** 10.1371/journal.pone.0359310

**Published:** 2026-10-09

**Authors:** Jie Wang, Yangqi Yin, Jiaqi Qu, Zhongbin Tao, Xuyang Chi, Xinyue Zhang

**Affiliations:** 1 Department of Pediatrics, The Second People’s Hospital of Gansu Province, Lanzhou, Gansu, China; 2 Department of Pediatrics, General Hospital of Fushun Mining Bureau of Liaoning Health Industry Group, Fushun, Liaoning, China; 3 College of Life Sciences, Wuhan University, Wuhan, Hubei, China; 4 Department of Pediatrics, The First Hospital of Lanzhou University, Lanzhou, Gansu, China; 5 Second Department of Internal Medicine, School of Medicine, University of Occupational and Environmental Health, Kitakyushu, Fukuoka, Japan; 6 Department of Hematology, Dalian Women and Children’s Medical Group, Dalian, Liaoning, China; Fondazione Policlinico Universitario Gemelli IRCCS, ITALY

## Abstract

**Background:**

Antiphospholipid syndrome is an autoimmune disorder characterized by recurrent thrombotic events and/or adverse pregnancy outcomes. Although three classification criteria—the 1999 Sapporo criteria, the Revised Sapporo criteria, and the 2023 ACR/EULAR antiphospholipid syndrome classification—have been developed primarily for research purposes to standardize patient selection, clinicians sometimes apply these criteria to aid in diagnostic decision-making. However, direct head-to-head studies comparing these three criteria remain scarce, and a comprehensive systematic review with network meta-analysis of their classification performance is lacking.

**Objectives:**

The primary objective is to conduct a traditional meta-analysis to evaluate and compare the performance (sensitivity, specificity, positive likelihood ratio [PLR], negative likelihood ratio [NLR]) of each individual classification criterion. Secondary objectives include: 1) Conducting a Bayesian network meta-analysis (NMA) to compare and rank the three criteria using superiority index (SI); and 2) Exploring sources of heterogeneity through predefined subgroup analyses (primary vs. secondary APS, thrombotic vs. obstetric APS).

**Methods:**

Adhering to the PRISMA-P statement, this protocol has been registered in PROSPERO (CRD420251074199). A comprehensive literature search will be performed in electronic databases (PubMed/MEDLINE, Embase, the Cochrane Library, Web of Science) and gray literature sources (ClinicalTrials.gov, abstracts from international congresses including ACR, EULAR) from their inception to October 2025. Eligible studies are classification performance studies (cross-sectional, cohort, or case-control) that assess at least one of the three criteria against the reference standard (expert clinical diagnosis by rheumatologists/APS specialists, based on comprehensive evaluation of clinical history, persistent antiphospholipid antibodies [aPLs] testing, and exclusion of alternative causes). The quality of included studies will be assessed using the QUADAS-2 tool. Bayesian NMA will be conducted in R version 4.3.0 with the RStan package, adopting a random-effects ANOVA model. Heterogeneity will be assessed via Cochrane’s Q test and the inconsistency index (I²), while publication bias will be evaluated using Deeks’ funnel plot asymmetry test.

## 1. Introduction

Antiphospholipid syndrome (APS) is an autoimmune disorder characterized by recurrent thrombotic events (arterial or venous) and/or pregnancy morbidity (e.g., unexplained fetal loss, early preeclampsia) in the presence of persistent antiphospholipid antibodies (aPLs), including anticardiolipin antibodies (aCL), anti-β_2_-glycoprotein I antibodies (aβ_2_GPI), and lupus anticoagulant (LAC) [1,2]. APS is categorized into primary and secondary forms, with the latter often associated with systemic lupus erythematosus (SLE) [3]. The pathogenesis of APS involves circulating aPLs binding to phospholipids and phospholipid-binding proteins on cell surfaces, which activates endothelial cells, platelets, and leukocytes, leading to vascular thrombotic events, obstetric complications, and promotion of other autoimmune and inflammatory conditions [4,5]. APS has an estimated global prevalence of 40–50 per 100,000 individuals, with an annual incidence rate of approximately 1–2 cases per 100,000 population [6,7]. The inaugural classification criteria for APS, established during the 1999 Sapporo consensus conference (Sapporo 1999) to standardize patient selection for research purposes, mandates that classification requires both at least one clinical manifestation (arterial/venous thrombosis or specific pregnancy complications) and persistent laboratory evidence of aPLs (either LAC and/or medium-to-high titer IgG/IgM aPLs), confirmed through repeat testing with a minimum six-week interval between assessments [8].

The 2006 Revised Sapporo criteria (Revised Sapporo) refined laboratory parameters by stipulating that aPLs must persist for at least 12 weeks, incorporating aβ_2_GPI encompassing both IgG and IgM as an independent diagnostic marker, and defining medium titers of aCL or aβ_2_GPI as exceeding 40 GPL or MPL or surpassing the 99th percentile [9].

Recently, the American College of Rheumatology (ACR) and the European League Against Rheumatism (EULAR) joined forces to issue the latest classification criteria (ACR 2023) for APS in 2023. The ACR 2023 require the concurrent presence of at least one clinical criterion and one laboratory criterion within a three-year period. Notably, the updated criteria adopt a weighted scoring system that quantifies both clinical features and laboratory parameters. Overall, these criteria encompass eight domains, six of which are clinical and two laboratory-based. In terms of clinical criteria, building on the original thromboembolic events and pathological pregnancies, the new criteria have added microangiopathy, cardiac valve lesions, and hematological system manifestations, which are further divided into 6 categories: venous thromboembolic events, arterial thromboembolic events, microangiopathy, pathological pregnancies, cardiac valve lesions, and hematological system damage. For laboratory criteria, risk stratification is performed based on the number of positive aPLs, their subtypes, titers, and the duration of LAC positivity [10].

Despite these updates, the classification performance (e.g., sensitivity, specificity, positive likelihood ratio [PLR], negative likelihood ratio [NLR]) of these criteria remains incompletely compared. Direct head-to-head studies of all three criteria are scarce, and existing evidence is fragmented, with most studies focusing on pairwise comparisons (e.g., Revised Sapporo vs. Sapporo 1999, Revised Sapporo vs. ACR 2023). Network meta-analysis (NMA) allows indirect comparisons of multiple interventions (here, classification criteria) when direct comparisons are limited, enabling ranking of their diagnostic accuracy. This systematic review and NMA aim to comprehensively evaluate and compare the diagnostic performance of the three criteria to inform clinical practice and future criteria updates.

It is important to acknowledge that all three criteria were developed for classification purposes in research settings, aiming to identify homogeneous patient populations for clinical trials and epidemiological studies, rather than serving as comprehensive diagnostic tools. Classification criteria typically prioritize high specificity to minimize false-positive inclusions in research cohorts, often at the expense of sensitivity [10]. However, clinicians frequently apply these criteria in practice to guide diagnostic reasoning, and understanding their classification performance in comparison to expert clinical judgment remains clinically relevant [11]. Therefore, this review evaluates the ‘classification performance’ of these criteria, while acknowledging that they should not be considered definitive diagnostic instruments.

## 2. Objectives

The primary objective is performing a traditional meta-analysis to assess and compare the classification performance (sensitivity, specificity, PLR, NLR and the area under the curve [AUC]) of each criterion individually.

Secondary objectives include:

1) Conduct a NMA to compare the relative classification performance of the three criteria (direct and indirect comparisons) and rank them by overall diagnostic utility (using superiority index [SI]).
2) Exploring heterogeneity by predefined subgroups (e.g., primary vs. secondary APS, thrombotic vs. obstetric APS).

## 3. Methods and analysis

The protocol is registered on the International Prospective Register for Systematic Reviews (PROSPERO, CRD420251074199). This protocol adheres to the Preferred Reporting Items for Systematic Review and Meta-Analysis Protocols (PRISMA-P) statement (see S1 Table for PRISMA-P checklist). The project commenced in June 2025, with results anticipated by May 2026.

### 3.1. Eligibility criteria

#### 3.1.1. Study design.

Diagnostic accuracy studies (cross-sectional, cohort, or case-control) that evaluate the performance of at least one of the three criteria (Sapporo 1999, Revised Sapporo, ACR 2023) against a reference standard for APS diagnosis. A systematic literature search was conducted based on the PICo framework: (1) Population: Patients of any age, gender, or ethnicity with suspected or confirmed APS (including primary APS, secondary APS associated with systemic lupus erythematosus or other autoimmune diseases, and obstetric APS). Studies including mixed populations (e.g., with and without APS) are eligible if data for the target population can be extracted; (2) Intervention: the Sapporo 1999, Revised Sapporo, or ACR 2023 classification criteria; (3) Comparison: reference standard; (4) Outcome: TP, FP, TN, and FN. Studies were included if they met the following criteria: (1) Evaluated at least one of the specified classification criteria (Sapporo 1999, Revised Sapporo, or ACR 2023); (2) Were published as full-text articles or conference abstracts; (3) Used standardized ELISA for detection of aCL and anti-β_2_GPI, and following the International Society of Thrombosis and Hemostasis (ISTH) guidelines, both dilute Russell’s viper venom time (dRVVT) and a sensitive activated partial thromboplastin time (APTT) were employed, combined with a three-step procedure (screening → mixing study → confirmation) for lupus anticoagulant (LAC) testing. Exclusion criteria comprised: (1) Case reports; (2) Studies lacking sufficient data to extract TP, FP, TN, and FN; (3) Studies not fully adhering to the above-mentioned standardized assays for aPLs. (4) The reference standard does not comply with the requirements. A comprehensive systematic literature search will be performed without any language restrictions. In cases of overlapping patient populations, the most recently published study with the largest sample size was included.

#### 3.1.2. Reference standard.

The reference standard will be defined as: Expert clinical diagnosis by at least two rheumatologists or APS specialists (with consensus required for final determination), based on comprehensive evaluation of clinical history, physical examination, laboratory findings (including aPL testing with persistence), and exclusion of alternative causes (e.g., inherited thrombophilias, other pregnancy complications, malignancies, infections). Importantly, to minimize incorporation bias, the expert panel’s diagnosis should be independent of the specific classification criteria being evaluated. The panel should consider all available clinical and laboratory information without systematically applying any single classification criterion as a diagnostic rule. This approach aims to preserve the independence between the index test and reference standard as required for diagnostic accuracy studies.

### 3.2. Information sources and search strategy

A comprehensive literature search will be conducted from database inception to October 2025. Electronic databases to be searched include PubMed/MEDLINE, Embase, the Cochrane Library (incorporating the Cochrane Database of Systematic Reviews and the Cochrane Central Register of Controlled Trials), and Web of Science. Additionally, gray literature sources will be searched, including ClinicalTrials.gov.

Search terms will include combinations of APS-related terms (e.g., “Antiphospholipid Syndrome”, “Syndrome, Antiphospholipid”, “Anti-Phospholipid Antibody Syndrome”, “Antibody Syndrome, Anti-Phospholipid”, “Anti Phospholipid Antibody Syndrome”, “Syndrome, Anti-Phospholipid Antibody”, “Anti-Phospholipid Syndrome”, “Anti Phospholipid Syndrome”, “Syndrome, Anti-Phospholipid”, “Antiphospholipid Antibody Syndrome”, “Antibody Syndrome, Antiphospholipid”, “Antiphospholipid Antibody Syndromes”, “Syndrome, Antiphospholipid Antibody”, “Hughes Syndrome”, “Syndrome, Hughes”) and classification criteria terms (e.g., “Sapporo”, “revised”, “Sydney”, “American College of Rheumatology”, “ACR”, “European League Against Rheumatism”, “EULAR”). An example search strategy for PubMed is provided in S2 Table. Reference lists of included studies and relevant systematic reviews will be hand-searched to identify additional studies.

### 3.3. Study selection and data extraction

A customized data extraction form will be employed in Microsoft Excel to facilitate structured data collection. Two reviewers (Y.Y. and J.W.) will independently screen titles and abstracts against eligibility criteria. Full texts of potentially eligible studies will be retrieved and assessed for inclusion. Disagreements will be resolved by consensus or consultation with a third reviewer (X.C.). The PRISMA flow diagram attached in Fig 1 illustrates the process of study selection. A predefined data extraction form will be used to systematically collect information from included studies. This will encompass study characteristics (first author, publication year, country, study design, sample size, follow-up duration), patient characteristics (age, gender, APS subtype [primary/secondary, thrombotic/obstetric], comorbidities), index test details (specific diagnostic criteria applied, methods for aPL testing including assays, cutoffs, and persistence assessment), reference standard details (definition of expert diagnosis, follow-up duration used for confirmation), and diagnostic accuracy data (true positives [TP], false positives [FP], true negatives [TN], false negatives [FN] for each criterion). Two reviewers (Y.Y. and J.W.) will perform data extraction independently, cross-check results for accuracy, and resolve discrepancies through consensus. For missing data, the corresponding study authors will be contacted via email, with up to three attempts made to obtain the required information.

**Fig 1:**
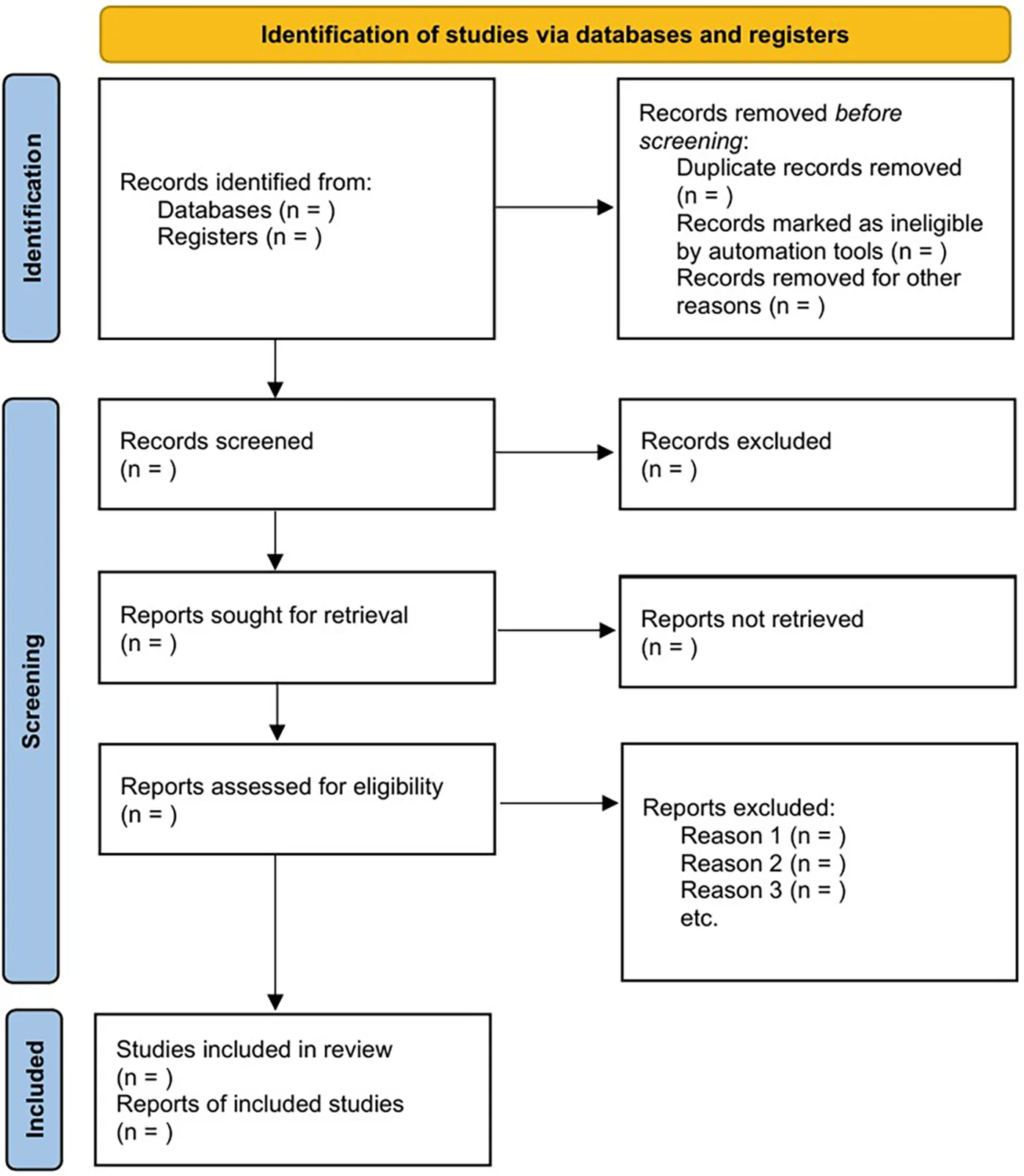
PRISMA flow diagram for the systematic review and meta- analysis protocol. Based on the PRISMA 2020 guidelines, this diagram outlines the identification, screening, and final inclusion of studies from databases and registers.

To assess the feasibility of the planned analyses, we conducted a preliminary search of PubMed, Embase, Cochrane Library and Web of Science in October 2025. This preliminary search identified 3180 potentially relevant records. After title/abstract screening and full-text review of the most relevant 37 articles, we identified 13 studies evaluating at least one of the three classification criteria against a reference standard. Specifically, 11 studies evaluated the Revised Sapporo criteria, 2 evaluated the Sapporo 1999 criteria, and 12 studies evaluated the ACR 2023 criteria. Based on these preliminary findings, we anticipate sufficient studies for pairwise meta-analyses, particularly for the Revised Sapporo and Sapporo 1999 criteria. The limited number of studies for the ACR 2023 criteria (n = 1) is acknowledged as a limitation, and the network meta-analysis will be interpreted with caution, with sensitivity analyses planned to assess the robustness of findings when combining data across criteria.

### 3.4. Dealing with missing required information

When required information is missing or unclear in studies, we will email the corresponding authors for further details. Should such information remain unobtainable, we will omit the relevant studies.

### 3.5. Quality assessment

The quality of included studies will be assessed using the Quality Assessment of Diagnostic Accuracy Studies-2 (QUADAS-2) tool [12], which evaluates four domains: (1) patient selection, (2) index test, (3) reference standard, and (4) flow and timing. Each domain is rated as “low”, “high”, or “unclear” risk of bias, and applicability concerns (whether the study population, index test, or reference standard are relevant to the review question) are also assessed. Assessment will be conducted independently by two reviewers (Y.Y. and J.W.), with disagreements resolved by consensus.

### 3.6. Data synthesis and analysis

Study data will be quantitatively synthesized only if the included studies demonstrate sufficient clinical and methodological similarity, and provided that a sufficient number of studies (≥2) report extractable and comparable outcome data for statistical pooling.

#### 3.6.1. Traditional Meta-analysis and systematic review.

The analysis will proceed as follows: First, the “metandi” and “midas” commands in STATA 17.0 will be used to implement a bivariate random-effects logistic regression model, from which pooled sensitivity, specificity, PLR, NLR, and the summary AUC will be derived. These estimates for sensitivity, specificity, PLR, and NLR, as well as their 95% CIs will then be presented in a forest plot. Finally, the hierarchical summary receiver operating characteristic (HSROC) curve will be calculated. Should significant clinical or methodological heterogeneity preclude a quantitative meta-analysis, a structured narrative synthesis will be conducted. This will involve a systematic presentation of the findings as follows: First, the characteristics and results of all included studies will be summarized in evidence tables. Second, the reported estimates of sensitivity, specificity, and other relevant metrics will be presented without statistical pooling. The findings will then be synthesized by describing the direction, magnitude, and consistency of the effects across studies, and by exploring potential explanations for the observed variations in results.

#### 3.6.2. Network meta-analysis.

The network meta-analysis will be performed within a Bayesian framework utilizing R version 4.3.0. Multiple comparisons of diagnostic tests will be performed using the ANOVA model developed by Nyaga et al [13]. This model estimates between-study random effects for the sensitivity and specificity of different tests, as well as within-study random effects for specific tests. A two-tailed p-value of less than 0.05 will be considered statistically significant. Furthermore, the three diagnostic criteria will be ranked according to the SI described by Deutsch et al [14]. This SI ranks tests by conferring higher weights upon those with strong performance in both sensitivity and specificity, while penalizing those that perform poorly on both or are highly imbalanced. Its values span from 0 to ∞. A result asymptotically approaching 1 denotes equivalent test performance, whereas a trend toward ∞ or 0 reflects progressively greater strengths or weaknesses, respectively. Bayesian analysis will be performed using the Rstan package. Markov Chain Monte Carlo (MCMC) sampling will be carried out with two chains, each consisting of 10,000 iterations, which includes a warm-up phase of 5,000 iterations and a thinning interval of 5. Default non-informative priors available in Rstan will be applied to reduce the impact of prior specifications on parameter estimates. Model convergence will be evaluated using the potential scale reduction factor (R-hat), where values less than 1.1 will be taken to indicate adequate convergence.

#### 3.6.3. Heterogeneity and subgroup analyses.

Statistical heterogeneity will be assessed using Cochrane’s Q test and the inconsistency index (I²). Predefined subgroup analyses evaluating the influence of APS subtype (primary vs. secondary; thrombotic vs. obstetric). Sensitivity analyses will exclude studies with high risk of bias or missing data to test the robustness of results.

#### 3.6.4. Handling variability in antiphospholipid antibody testing.

Variability in aPL testing across laboratories and studies represents a major source of potential heterogeneity that we will address through multiple strategies: Firstly, as specified in Section 3.1.1, we will only include studies that employ ISTH-recommended LAC testing protocols and standardized ELISA methods for aCL and anti-β2GPI, with documented assay thresholds (e.g., 99th percentile, GPL/MPL units). Secondly, for each included study, we will extract detailed information on assay methods (ELISA manufacturer, cutoffs), LAC testing protocols, and persistence criteria (time intervals, repeat testing). Thirdly, where sufficient data exist, we will conduct subgroup analyses stratifying by aPL assay characteristics to explore whether these factors influence classification performance.

#### 3.6.5. Publication bias.

The assessment of publication bias will be performed using Deeks’ funnel plot asymmetry test when the number of included trials reaches ten [15].

### 3.7. Study status and timeline

This systematic review and network meta-analysis is currently in its preliminary stage, and no results have been generated to date. Only a preliminary search of PubMed, Embase, Cochrane Library and Web of Science have been conducted as a pilot; formal searches of other databases have not yet been initiated, and no data extraction has been performed. The planned timeline for the study is outlined below:

- Literature search and screening: Expected to be completed by January 2026.
- Data extraction: Planned to be concluded by March 2026.
- Data analysis and interpretation: Anticipated to be finalized by May 2026.
- Manuscript preparation and submission: Expected by e August 2026.

All phases of the study will be carried out independently by two reviewers to ensure accuracy and consistency. Any future amendments to the study protocol will be documented and promptly updated in the PROSPERO registry.

### 3.8. Ethics statements

This review will synthesize data from published studies, so ethical approval is not required.

## 4. Grading of recommendations

We will use the GRADE system to assess the overall strength of the evidence, which will be classified into one of four certainty levels: high, moderate, low, or very low [16].

## 5. Dissemination

Results will be submitted for publication in a peer-reviewed journal or presented at relevant conferences (e.g., ACR, EULAR).

## 6. Limitations of this study

First, the three classification criteria were developed for research classification rather than for clinical diagnosis. Evaluating their “classification performance” against an expert clinical reference standard carries inherent conceptual challenges, which may potentially introduce bias. Although we have designed our reference standard to minimise this risk, complete independence from the criteria cannot be guaranteed. Second, APS is a relatively rare disease, with an estimated prevalence of 40–50 per 100,000 individuals. For the 1999 criteria, the number of validation studies is limited (n = 2 in our preliminary search), which may reduce the statistical power of comparative analyses, particularly for these criteria. Third, the lack of standardisation in aPL assays across laboratories and studies represents a major source of clinical heterogeneity. We will systematically assess this heterogeneity and, where feasible, adjust for it through predefined subgroup and sensitivity analyses. Fourth, incomplete data on key classification metrics (true positives, false positives, true negatives, and false negatives) may necessitate data imputation or exclusion of certain studies, potentially introducing selection bias. We will contact study authors to obtain missing data and will clearly report the proportion of studies for which complete data were available.

## Supporting information

S1 Table PRISMA-P 2015 checklist. This checklist outlines the recommended items to address in the systematic review protocol, detailing the item number, checklist item, and corresponding page numbers.
(PDF)

S2 Table Example PubMed search strategy. The table presents an example of the PubMed search strategy used to identify studies related to antiphospholipid syndrome. The search was conducted through the native PubMed interface on October 12, 2025.
(PDF)
