# Supplementary material for: Systematic review and network meta-analysis of performance of the Sapporo criteria, the Revised Sapporo criteria, and the 2023 ACR/EULAR APS classification criteria for patients with antiphospholipid syndrome: A protocol
Source: PLoS One. 2026 Oct 9;21(10):e0359310. doi: 10.1371/journal.pone.0359310 (PMC13652260; doi:10.1371/journal.pone.0359310)
Supplement: Supplementary file 2 [file pone.0359310.s002.pdf]

Supplemental Material 2: Example PubMed Search Strategy.

Pubmed via native interface on October 12, 2025

Search number	Query	Results
#1	"Antiphospholipid Syndrome"[Mesh]	9922
#2	Syndrome, Antiphospholipid[Title/Abstract] OR Anti-Phospholipid Antibody Syndrome[Title/Abstract] OR Antibody Syndrome, Anti-Phospholipid[Title/Abstract] OR Anti Phospholipid Antibody Syndrome[Title/Abstract] OR Syndrome, Anti-Phospholipid Antibody[Title/Abstract] OR Anti-Phospholipid Syndrome[Title/Abstract] OR Anti Phospholipid Syndrome[Title/Abstract] OR Syndrome, Anti-Phospholipid[Title/Abstract] OR Antiphospholipid Antibody Syndrome[Title/Abstract] OR Antibody Syndrome, Antiphospholipid[Title/Abstract] OR Antiphospholipid Antibody Syndromes[Title/Abstract] OR Syndrome, Antiphospholipid Antibody[Title/Abstract] OR Hughes Syndrome[Title/Abstract] OR Syndrome, Hughes[Title/Abstract]	2753
#3	#1 OR #2	11016
#4	American College of Rheumatology[Title/Abstract] OR ACR[Title/Abstract] OR European League Against Rheumatism[Title/Abstract] OR EULAR[Title/Abstract] OR Sapporo[Title/Abstract] OR Sydney[Title/Abstract] OR revised[Title/Abstract]	134564
#5	#3 AND #4	403
